# Rudolf Buchheim Award 2026: Toward a ToxAtlas of carbon-based nanomaterials: single-cell RNA sequencing reveals initiating cell circuits in pulmonary inflammation

**DOI:** 10.1007/s00210-026-05170-7

**Published:** 2026-03-18

**Authors:** Carola Voss

**Affiliations:** 1 Hannover Medical School, Clinic for Cardiac, Thoracic, Transplantation and Vascular Surgery, Leibniz Research Laboratories for Biotechnology and Artificial Organs (LEBAO), Biomedical Research in Endstage and Obstructive Lung Disease Hannover (BREATH), German Center for Lung Research (DZL), 30625 Hannover, Germany; 2 Institute of Lung Health and Immunity (LHI), Comprehensive Pneumology Center (CPC), Helmholtz Munich, German Center for Lung Research (DZL), 81377 Munich, Germany

**Keywords:** Carbon-based nanomaterials, Single-cell RNA sequencing, Pulmonary inflammation, Mode of actions, ToxAtlas

## Abstract

The Rudolf Buchheim Award of the German Society for Experimental and Clinical Pharmacology and Toxicology (DGPT) honors outstanding contributions by early-career scientists in pharmacology and toxicology. The 2026 award recognizes research that advances mechanistic understanding of the initiation of respiratory inflammation by carbon-based nanomaterials (CBN). While pulmonary inflammation is recognized as an early adverse but generic response to inhaled particles, only little is known about the initiating events and involved cell types. Carola Voss systematically dissected cell type–specific responses to soot-like carbon nanoparticles, flexible double-walled carbon nanotubes, and needle-like multi-walled carbon nanotubes known for their asbestos-like toxicity at single-cell resolution in the lab of Dr. Tobias Stoeger at Helmholtz Munich. Using advanced single-cell transcriptomics of CBN-exposed mouse lungs, this research establishes a toxicological atlas that links physicochemical CBN properties to cell-specific inflammatory initiation, cellular stress responses, and immune cell recruitment. The findings provide a conceptual framework for mode of action-based hazard assessment and support the rational design of safer nanomaterials along with the development of specific in vitro assays.

## Short communication

The rapid expansion of carbon-based nanomaterials (CBN), particularly carbon nanotubes (CNT), has generated unprecedented opportunities in electronics and materials science while simultaneously raising concerns regarding their potential adverse biological effects (Hill et al. 2023; Poland et al. 2008). Bulk readouts often fail to capture subtle, cell type- and material–specific stress responses that may ultimately drive inflammation, tissue remodeling, or chronic toxicity in the respiratory system. In contrast, single-cell RNA sequencing (scRNAseq) is well established in the biomedical field, for example, to advance our understanding of lung regeneration (Strunz et al. 2020). Consequently, there is a growing need for mechanistic frameworks that connect nanomaterial properties with defined biological response pathways at cellular resolution.

The Rudolf Buchheim Award 2026 recognizes Carola Voss for a comprehensive investigation into the cellular and molecular mechanisms underlying the initiation of CBN-induced respiratory inflammation, conducted in Dr. Tobias Stoeger’s lab at Helmholtz Munich and published 2025 in ACS Nano (Voss et al. 2025). This work introduces a single-cell–based toxicological atlas that systematically maps how a set of three structurally distinct CBN, carbon nanoparticles (CNP), double-walled CNT (DWCNT), and multi-walled CNT (MWCNT), engage cellular stress, inflammatory, and immune signaling programs across heterogeneous cell populations.

A central innovation of this study lies in scRNAseq with classical protein-based response analyses at the early initiating state of the inflammatory response. Rather than treating exposed cell populations as homogeneous entities, the approach resolves transcriptional responses at the level of individual cells, enabling the identification of biologically decisive response states. This strategy revealed that even at the chosen doses, all CBN induce a comparable acute inflammatory response when quantified by recruited neutrophil numbers in the airspace; however, they elicit highly material- and cell type–specific, inflammatory transcriptional and cellular programs. Fibroblasts, epithelial cells, and macrophage populations were found to differ markedly in their sensitivity and response profiles. In particular, subsets of fibroblasts emerged as key sentinel cells that translate CBN-induced stress into paracrine inflammatory signaling. These fibroblast subpopulations upregulated chemokines such as Ccl2 and Ccl7, which are known drivers of monocyte recruitment and macrophage activation. This finding repositions fibroblasts from passive structural cells to active regulators of nanomaterial-induced interstitial inflammatory cascades.

**Fig. 1:**
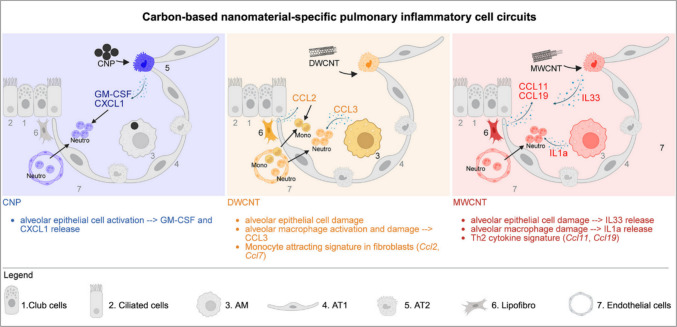
CBN-specific pulmonary inflammatory cell circuits. Reused under CC-BY 4.0 from ACS Nano (Voss et al. 2025)

The study further demonstrates that transcriptional response patterns are tightly linked to CBN physicochemical characteristics, particularly aspect ratio, rigidity, and aggregation behavior. By correlating these material features with pathway-level transcriptional signatures, cytokine profiling, and tissue analyses, the work moves beyond descriptive toxicology toward a predictive, mechanism-based paradigm. Notably, inflammatory signaling was not solely associated with overt cytotoxicity but could be triggered at sublethal exposure levels, underscoring the importance of sensitive molecular endpoints for nanosafety evaluation. The study by Voss et al. demonstrates that, depending on the type of CBN exposure to the lungs, the neutrophil recruitment observed across all materials is initiated through distinct cellular mechanisms. Specifically, recruitment is triggered either by alveolar epithelial cells via the release of CXCL1 (CNP), by activated alveolar macrophages (AM) through the secretion of CCL3 (DWCNT), or by damaged AM releasing IL-1α (MWCNT) (Fig. 1).

Single-cell profiling of immune populations revealed that macrophages and monocytes adopt distinct activation states depending on the upstream stromal and epithelial signals induced by CBN. The fibroblast-derived chemokine axis emerged as a central amplifier of immune cell recruitment, suggesting that stromal–immune crosstalk is a critical determinant of nanomaterial-induced inflammation. These findings provide mechanistic insight into how localized cellular stress responses can scale into tissue-level inflammatory outcomes, a process highly relevant for chronic exposure scenarios.

By establishing a single-cell toxicological atlas, this work delivers an important conceptual advance for experimental particle toxicology. It demonstrates that adverse effects of CBN cannot be fully understood without resolving cellular heterogeneity and intercellular communication networks. The approach offers a blueprint for future studies aiming to classify CBN based on mode of action rather than solely on classical toxicity endpoints. Importantly, it presents a critical breakthrough in enabling robust and mechanistically informative cell-based assays for CBN reflecting in vivo outcomes.

From a translational perspective, the findings support the rational engineering of safer nanomaterials by identifying physicochemical features that minimize activation of harmful stress and inflammatory pathways. Moreover, the atlas framework may be adapted to other classes of advanced materials and xenobiotics, thereby extending its relevance beyond nanotoxicology.

The research recognized by the Rudolf Buchheim Prize 2026 provides a mechanistic, single-cell–resolved view of how CBN interact with the respiratory system. By linking nanomaterial properties to defined transcriptional programs in epithelial, stromal, and immune cells, it advances nanosafety science from phenomenology toward prediction. This work exemplifies how modern molecular technologies can refine toxicological risk assessment and aligns closely with the DGPT’s mission to promote mechanism-based experimental pharmacology and toxicology.

## Data Availability

There was no data used for this article.

## References

[CR1] Hill W, Lim EL, Weeden CE, Lee C, Augustine M, Chen K, Kuan F-C, Marongiu F, Evans EJ, Moore DA et al (2023) Lung adenocarcinoma promotion by air pollutants. Nature 616:159–167 37020004 10.1038/s41586-023-05874-3 PMC7614604

[CR2] Poland CA, Duffin R, Kinloch I, Maynard A, Wallace WAH, Seaton A, Stone V, Brown S, MacNee W, Donaldson K (2008) Carbon nanotubes introduced into the abdominal cavity of mice show asbestos-like pathogenicity in a pilot study. Nat Nanotechnol 3:423–428 18654567 10.1038/nnano.2008.111

[CR3] Strunz M, Simon LM, Ansari M, Kathiriya JJ, Angelidis I, Mayr CH, Tsidiridis G, Lange M, Mattner LF, Yee M et al (2020) Alveolar regeneration through a Krt8+ transitional stem cell state that persists in human lung fibrosis. Nat Commun 11:3559 32678092 10.1038/s41467-020-17358-3 PMC7366678

[CR4] Voss C, Han L, Ansari M, Strunz M, Haefner V, Angelidis I, Mayr CH, Berthing T, Zhou Q, Guenther EM et al (2025) Toward a ToxAtlas of carbon-based nanomaterials: single-cell RNA sequencing reveals initiating cell circuits in pulmonary inflammation. ACS Nano 19:39139–39156 41183169 10.1021/acsnano.5c12054 PMC12632174

